# Efficacy and safety of immune checkpoint inhibitor rechallenge in individuals with hepatocellular carcinoma

**DOI:** 10.1016/j.jhepr.2022.100620

**Published:** 2022-10-27

**Authors:** Bernhard Scheiner, Daniel Roessler, Samuel Phen, Mir Lim, Katharina Pomej, Tiziana Pressiani, Antonella Cammarota, Thorben W. Fründt, Johann von Felden, Kornelius Schulze, Vera Himmelsbach, Fabian Finkelmeier, Ansgar Deibel, Alexander R. Siebenhüner, Kateryna Shmanko, Pompilia Radu, Birgit Schwacha-Eipper, Matthias P. Ebert, Andreas Teufel, Angela Djanani, Florian Hucke, Lorenz Balcar, Alexander B. Philipp, David Hsiehchen, Marino Venerito, Friedrich Sinner, Michael Trauner, Antonio D'Alessio, Claudia A.M. Fulgenzi, David J. Pinato, Markus Peck-Radosavljevic, Jean-François Dufour, Arndt Weinmann, Andreas E. Kremer, Amit G. Singal, Enrico N. De Toni, Lorenza Rimassa, Matthias Pinter

**Affiliations:** 1 Division of Gastroenterology and Hepatology, Department of Internal Medicine III, Medical University of Vienna, Vienna, Austria; 2 Liver Cancer (HCC) Study Group Vienna, Division of Gastroenterology and Hepatology, Department of Internal Medicine III, Medical University of Vienna, Vienna, Austria; 3 Department of Medicine II, University Hospital, LMU Munich, Munich, 81377, Germany; 4 Department of Medicine, UT Southwestern Medical Center, Dallas TX, USA; 5 Medical Oncology and Hematology Unit, Humanitas Cancer Center, IRCCS Humanitas Research Hospital, Via Manzoni 56, 20089 Rozzano (Milan), Italy; 6 Department of Biomedical Sciences, Humanitas University, Via Rita Levi Montalcini 4, 20072 Pieve Emanuele (Milan), Italy; 7 1. Department of Internal Medicine, Gastroenterology & Hepatology, University Medical Center Hamburg-Eppendorf, Hamburg, Germany; 8 Department of Gastroenterology, Hepatology and Endocrinology, University Hospital Frankfurt, Frankfurt/Main, Germany; 9 Department of Hepatology and Gastroenterology, University Hospital Zurich and University Zurich, Zurich, Switzerland; 10 Department of Medical Oncology and Hematology, University Hospital Zurich and University Zurich, Zurich, Switzerland; 11 Department of Medical Oncology and Hematology, Cantonal Hospital Schaffhausen, Schaffhausen, Switzerland; 12 Department of Internal Medicine I, University Medical Center of the Johannes Gutenberg University Mainz, Mainz, Germany; 13 Hepatology-Department of Biomedical Research, University of Bern, Bern, Switzerland; 14 Department of Visceral Surgery and Medicine, Inselspital, University of Bern, Bern, Switzerland; 15 Department of Internal Medicine II, Medical Faculty Mannheim, Heidelberg University, Mannheim, Germany; 16 Clinical Cooperation Unit Healthy Metabolism, Center for Preventive Medicine and Digital Health Baden-Württemberg (CPDBW), Medical Faculty Mannheim, Heidelberg University, Mannheim, Germany; 17 DKFZ-Hector Cancer Institute at University Medical Center Mannheim, Mannheim, Germany; 18 Department of Internal Medicine II, Division of Hepatology, Medical Faculty Mannheim, Heidelberg University, Mannheim, Germany; 19 Department of Internal Medicine I, Division of Gastroenterology, Hepatology, Endocrinology and Metabolism, Medical University of Innsbruck, Innsbruck, Austria; 20 Internal Medicine and Gastroenterology (IMuG), Including Centralized Emergency Service (ZAE), Klinikum Klagenfurt am Wörthersee, Klagenfurt, Austria; 21 Department of Gastroenterology, Hepatology and Infectious Diseases, Otto-Von Guericke University Hospital, 39120 Magdeburg, Germany; 22 Department of Surgery & Cancer, Imperial College London, Hammersmith Hospital, London, UK; 23 Department of Medical Oncology, University Campus Bio-Medico of Rome, Italy; 24 Division of Oncology, Department of Translational Medicine, University of Piemonte Orientale, Novara, Italy; 25 Department of Medicine 1, Friedrich-Alexander-University Erlangen-Nürnberg, Erlangen, Germany

**Keywords:** Liver cancer, Immunotherapy, Systemic therapy, Immune checkpoint blocker, BOR, best overall response, CR, complete response, DCR, disease control rate, HCC, hepatocellular carcinoma, ICI, immune checkpoint inhibitor, NE, not evaluable, ORR, objective response rate, OS, overall survival, PD, progressive disease, PR, partial response, SD, stable disease, TRAEs, treatment-related adverse events, TTP, time-to-progression

## Abstract

**Background & Aims:**

We investigated the efficacy and safety of immune checkpoint inhibitor (ICI) rechallenge in patients with hepatocellular carcinoma (HCC) who received ICI-based therapies in a previous systemic line.

**Methods:**

In this international, retrospective multicenter study, patients with HCC who received at least two lines of ICI-based therapies (ICI-1, ICI-2) at 14 institutions were eligible. The main outcomes included best overall response and treatment-related adverse events.

**Results:**

Of 994 ICI-treated patients screened, a total of 58 patients (male, n = 41; 71%) with a mean age of 65.0±9.0 years were included. Median systemic treatment lines of ICI-1 and ICI-2 were 1 (range, 1-4) and 3 (range, 2-9), respectively. ICI-based therapies used at ICI-1 and ICI-2 included ICI alone (ICI-1, n = 26, 45%; ICI-2, n = 4, 7%), dual ICI regimens (n = 1, 2%; n = 12, 21%), or ICI combined with targeted therapies/anti-VEGF (n = 31, 53%; n = 42, 72%). Most patients discontinued ICI-1 due to progression (n = 52, 90%). Objective response rate was 22% at ICI-1 and 26% at ICI-2. Responses at ICI-2 were also seen in patients who had progressive disease as best overall response at ICI-1 (n = 11/21; 52%). Median time-to-progression at ICI-1 and ICI-2 was 5.4 (95% CI 3.0-7.7) months and 5.2 (95% CI 3.3-7.0) months, respectively. Treatment-related adverse events of grade 3-4 at ICI-1 and ICI-2 were observed in 9 (16%) and 10 (17%) patients, respectively.

**Conclusions:**

ICI rechallenge was safe and resulted in a treatment benefit in a meaningful proportion of patients with HCC. These data provide a rationale for investigating ICI-based regimens in patients who progressed on first-line immunotherapy in prospective trials.

**Impact and implications:**

Therapeutic sequencing after first-line immune checkpoint inhibitor (ICI)-based therapy for advanced hepatocellular carcinoma (HCC) remains a challenge as no available second-line treatment options have been studied in immunotherapy-pretreated patients. Particularly, the role of ICI rechallenge in patients with HCC is unclear, as data from prospective trials are lacking. We investigated the efficacy and safety of ICI-based regimens in patients with HCC pretreated with immunotherapy in a retrospective, international, multicenter study. Our data provide the rationale for prospective trials investigating the role of ICI-based regimens in patients who have progressed on first-line immunotherapy.

## Highlights

• ICI rechallenge is uncommon in clinical practice – we screened 994 patients at 14 institutions and identified 58 cases of ICI rechallenge.
• ORR, DCR, and median TTP were comparable between first and second ICI-based treatment.
• Objective responses were seen in patients with progressive disease as best radiological response during first ICI-based treatment.
• They were also observed in those receiving atezolizumab/bevacizumab as a prior ICI regimen.
• ICI rechallenge was safe in our cohort and high-grade (grade 3-4) treatment-related adverse events were uncommon.

## Graphical abstract

**Figure:**
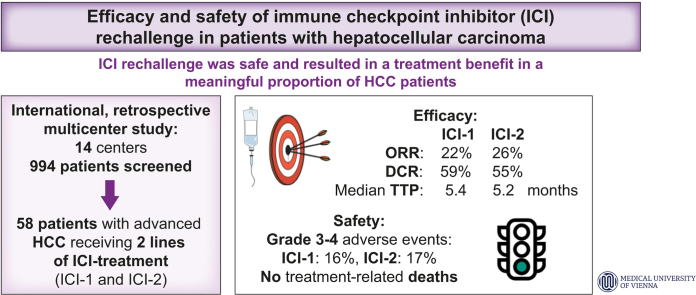

## Introduction

Hepatocellular carcinoma (HCC) is the most common primary liver cancer and a leading cause of cancer-related mortality worldwide.^1^ Most patients become candidates for systemic therapy at some point during the course of the disease. The systemic treatment landscape of HCC has changed rapidly over the last years.^2^ Several immune checkpoint inhibitors (ICIs) have been added to the treatment armamentarium in the United States after receiving conditional approval for sorafenib-pretreated patients following promising phase II data.^3^ The combination of atezolizumab/bevacizumab was the first ICI-based regimen to meet its primary survival endpoints *vs.* sorafenib in a phase III trial, and consequently became the standard of care in systemic front-line treatment.^2^^,^^4^^,^^5^ Only recently, the combination of durvalumab/tremelimumab was shown to be superior to sorafenib in terms of overall survival in a phase III trial, and durvalumab alone was non-inferior to sorafenib;^6^ thus, both will likely be added as additional first-line options upon approval.^7^

Sequencing after first-line immunotherapy is currently empirical in HCC and largely based on clinical characteristics and toxicity profiles, as well as local regulations and drug availabilities.^2^^,^^8^ The role of subsequent ICI use in ICI-pretreated patients with HCC is unclear, as data from prospective trials are lacking. Successful ICI rechallenge in a subset of patients has been reported in other solid tumors, including melanoma^9^ and renal cell carcinoma,^10^^,^^11^ providing the rationale for evaluating this strategy also in patients with HCC.

In this international, retrospective, multicenter study, we investigated the efficacy and safety of ICI-based regimens in patients with HCC who had received ICIs in a previous line of systemic therapy.

## Patients and methods

### Patients

In this international, retrospective multicenter study, patients with histologically or radiologically diagnosed HCC who received at least two lines of ICI-based therapies (ICI-1, ICI-2) at 14 institutions in Austria, Germany, Italy, Switzerland, United Kingdom, and the United States were considered. Patients who received two lines of different ICIs alone or as combination therapy and patients who received the same ICI at ICI-1 and ICI-2 but with a different combination partner were eligible. Patients were allowed to receive one or more treatments between ICI-1 and ICI-2.

Patients who received ICIs in a curative setting as (neo)adjuvant treatment before/after resection or ablation, and patients who received loco-regional therapies as the main treatment but in combination with ICIs were not included. The retrospective analysis was approved by the Ethics Committee of the Medical University of Vienna.

### Assessments and outcomes

Main outcomes included investigator-assessed best overall response (BOR) and treatment-related adverse events (TRAEs) according to Common Terminology Criteria for Adverse Events version 5.0. Objective response rate (ORR) was defined as the proportion of patients with complete response (CR) or partial response (PR) as BOR. Disease control rate (DCR) was defined as the proportion of patients achieving CR/PR or stable disease (SD) as BOR. Further outcomes included time-to-progression (TTP) as well as overall survival (OS).

### Statistical analysis

**Fig. 1:**
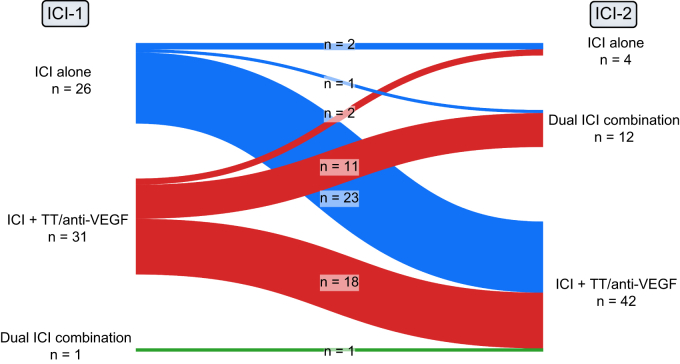
Type of immune checkpoint inhibitor regimen used at first and second line of ICI treatment. ICI, immune checkpoint inhibitor; ICI-1, first line of ICI treatment; ICI-2, second line of ICI treatment; TT, targeted therapy, VEGF, vascular endothelial growth factor.

**Fig. 2:**
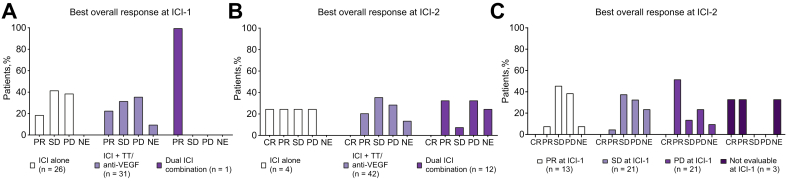
BOR at ICI-1 and ICI-2 line in selected patient populations. (A) BOR at ICI-1 according to type of therapy received at ICI-1, (B) BOR at ICI-2 according to type of therapy received at ICI-2, and (C) BOR at ICI-2 according to BOR at ICI-1. BOR, best overall response; CR, complete response; ICI, immune checkpoint inhibitor; ICI-1, first line of ICI treatment; ICI-2, second line of ICI treatment; NE, not evaluable; PD, progressive disease; PR, partial response; SD, stable disease; TT, targeted therapy, VEGF, vascular endothelial growth factor.

Data on baseline characteristics, radiological tumor evaluation, and TRAEs were summarized using descriptive statistics. Median duration of treatment was defined as time from the date of treatment initiation until the date of last administration; patients who were still receiving immunotherapy at data cut-off were censored. Patients who had at least one follow-up imaging were evaluable for assessment of BOR and TTP. TTP was defined as the time from the date of treatment initiation until the date of first radiologically confirmed tumor progression; patients without radiologically confirmed tumor progression were censored at the date of last imaging. OS was defined as the time from treatment start until date of death; patients who were still alive were censored at the date of last contact. Survival curves were calculated using the Kaplan-Meier method. Statistical analyses were performed using IBM SPSS Statistics version 26.0 (SPSS Inc., Chicago, IL). Fig. 1 was created using the software sankeyMATIC freely available at https://sankeymatic.com and Fig. 2 was created using GraphPad Prism 9 (GraphPad Software, LLC, San Diego, US). Median follow-up time was calculated using the reverse Kaplan-Meier method.

## Results

### Patients

Of the 994 ICI-treated patients with HCC screened, 58 (6%) patients were put on another ICI-based regimen between March 2019 and March 2022 after prior ICI discontinuation and were thus included in this analysis. Detailed patient characteristics at start of ICI-1 and ICI-2 are displayed in Table 1. Most patients had well-preserved liver function (Child-Pugh A) at ICI-1 (n = 55, 95%) and ICI-2 (n = 50, 86%). The majority had BCLC stage C at ICI-1 (n = 44, 76%) and ICI-2 (n = 48, 83%). Forty-two (72%) patients received any prior HCC treatment before immunotherapy. Most patients received ICI-1 as first- (n = 36, 62%) and ICI-2 as second- (n = 29, 50%) line systemic therapy. Seventeen patients (29%) received at least one systemic line between ICI-1 and ICI-2. ICI-based regimens used at ICI-1 and ICI-2 are shown in Table S1 and Fig. 1. Fifty-two patients (90%) discontinued ICI-1 due to radiological progression, 4 (7%) because of adverse events, one (2%) due to clinical progression, and another one (2%) due to patient preference. Median duration of ICI-1 and ICI-2 was 5.4 (95% CI 4.3-6.5) months and 3.6 (95% CI 2.4-4.9) months, respectively. Median duration from ICI-1 discontinuation to ICI-2 initiation was 1.3 (95% CI 0.4-2.1) months.

**Table 1:** **Patient characteristics at ICI-1 and ICI-2**.

	ICI-1 (n = 58; 100%)	ICI-2 (n = 58; 100%)
Age (years), mean ± SD	65.0±9.0	68.2±9.4
Sex, male	41 (71%)	—
Viral etiology	24 (41%)	—
Child-Pugh stage		
A	55 (95%)	50 (86%)
B	2 (3%)	8 (14%)
C	1 (2%)	0
ECOG PS∗^,^ ≥1	10 (17%)	24 (41%)
Treatment prior to ICI-1		
Surgery	25 (43%)	—
Ablation	7 (12%)	—
Loco-regional (TACE, SIRT, radiation)	20 (34%)	—
Systemic	22 (38%)	—
Macrovascular invasion	21 (36%)	21 (36%)
Extrahepatic metastases	29 (50%)	34 (59%)
BCLC stage		
B	13 (22%)	10 (17%)
C	44 (76%)	48 (83%)
D	1 (2%)	0
Alpha-fetoprotein (IU/ml)∗∗, median (IQR)	54.3 (5.7-902.2)	182.2 (5.8-7,907.4)
Line of ICI therapy, median (range)	1 (1-4)	3 (2-9)
Type of ICI regimen		
ICI alone	26 (45%)	4 (7%)
Dual ICI combination	1 (2%)	12 (21%)
ICI plus TT/anti-VEGF	31 (53%)	42 (72%)
Reason for discontinuation of ICI-1		
Radiological progression	52 (90%)	—
Toxicity	4 (7%)	—
Other	2 (3%)	—

BCLC, Barcelona-Clinic Liver Cancer; ECOG PS, Eastern Cooperative Oncology Group Performance Status; ICI, immune checkpoint inhibitor; ICI-1, first line of ICI treatment; ICI-2, second line of ICI treatment; SIRT, selective internal radiotherapy; TACE, transarterial chemoembolization; TT, targeted therapy; VEGF, vascular endothelial growth factor.

∗ Missing ICI-1: n = 1.

∗∗ Missing ICI-1: n = 2 and ICI-2: n = 4.

### Efficacy

Median estimated follow-up from ICI-1 was 25.1 (95% CI 20.8-29.4) months. Twenty-six patients (45%) died during the observation period. Median OS from initiation of systemic first-line, start of ICI-1, and start of ICI-2 was 47.0 (95% CI 39.9-54.2) months, 39.8 (95% CI 33.7-45.9) months, and 12.0 (95% CI 7.5-16.5) months, respectively. BOR at ICI-1 was CR/PR/SD/progressive disease (PD)/not evaluable in 0 (0%)/13 (22%)/21 (36%)/21 (36%)/3 (5%) patients, corresponding to an ORR and DCR of 22% and 59%, respectively. BOR at ICI-2 was CR/PR/SD/PD/not evaluable in 1 (2%)/14 (24%)/17 (29%)/17 (29%)/9 (16%) patients, corresponding to an ORR and DCR of 26% and 55%, respectively (Table 2). One patient (2%) had an objective response at both ICI-1 and ICI-2.

**Table 2:** **Main efficacy outcomes at ICI-1 and ICI-2**.

	ICI-1	ICI-2
Best overall response		
CR	0	1 (2%)
PR	13 (22%)	14 (24%)
SD	21 (36%)	17 (29%)
PD	21 (36%)	17 (29%)
N/E	3 (5%)	9 (16%)
ORR (CR+PR)	13 (22%)	15 (26%)
DCR (CR+PR+SD)	34 (59%)	32 (55%)
TTP, median (95%CI)	5.4 (3.0-7.7) months	5.2 (3.3-7.0) months

CR, complete response; DCR, disease control rate; ICI-1, first line of ICI treatment; ICI-2, second line of ICI treatment; N/E, not evaluable; ORR, objective response rate; PD, progressive disease; PR, partial response; SD, stable disease; TTP, time-to-progression.

Median TTP was 5.4 (95% CI 3.0-7.7) months (ICI-1) and 5.2 (95% CI 3.3-7.0) months (ICI-2), respectively (Table 2). Responses at ICI-2 were also seen in patients who had PD as BOR at ICI-1 (n = 11/21; 52%), and who received ICI monotherapy at ICI-2 (n = 2/4; 50%) (Fig. 2). Characteristics of individual patients who achieved a CR/PR at ICI-2 are displayed in Table 3.

**Table 3:** **Details of patients with a response at ICI-2**.

Patient #	Type of ICI-1	Line of ICI-1	BOR at ICI-1	Reason for discontinuation of ICI-1	Time between ICI-1 and ICI-2 (months)	Type of ICI-2	Line of ICI-2	BOR at ICI-2
1	Pembrolizumab	3	PR	Progression	5.4	Atezolizumab/bevacizumab	6	PR
2	Atezolizumab/bevacizumab	1	N/E	Adverse event	0.5	Pembrolizumab	2	CR
3	Nivolumab	2	PD	Adverse event	0.9	Pembrolizumab	3	PR
4	Pembrolizumab	4	PD	Progression	6.5	Atezolizumab/bevacizumab	6	PR
5	Pembrolizumab	1	PD	Progression	3.3	Atezolizumab/bevacizumab	2	PR
6	Nivolumab	3	PD	Progression	22.8	Atezolizumab/bevacizumab	6	PR
7	Pembrolizumab	1	PD	Progression	1.1	Atezolizumab/bevacizumab	2	PR
8	Pembrolizumab	2	PD	Progression	5.6	Pembrolizumab/lenvatinib	4	PR
9	Nivolumab	2	PD	Progression	18.8	Atezolizumab/bevacizumab	4	PR
10	Nivolumab/lenvatinib	1	SD	Adverse event	0.5	Nivolumab/ipilimumab	2	PR
11	Atezolizumab/bevacizumab	2	PD	Progression	0.7	Nivolumab/ipilimumab	3	PR
12	Nivolumab/lenvatinib	1	PD	Progression	0.5	Nivolumab/ipilimumab	2	PR
13	Atezolizumab/Bevacizumab	2	PD	Progression	10.2	Nivolumab/ipilimumab	5	PR
14	Pembrolizumab/bavituximab	1	PD	Progression	1.1	Pembrolizumab/bevacizumab	2	PR
15	Pembrolizumab/bavituximab	1	N/E	Patient’s preference	0.7	Atezolizumab/bevacizumab	2	PR

BOR, best overall response; CR, complete response; ICI-1, first line of ICI treatment; ICI-2, second line of ICI treatment; N/E, not evaluable; PD, progressive disease; PR, partial response; SD, stable disease.

In the subgroup of patients receiving atezolizumab/bevacizumab at ICI-1 (n = 17; 29%), 2 (12%) and 15 (88%) patients were treated with ICI monotherapy and combinatorial regimens at ICI-2, respectively (Table S2). The ORR and DCR at ICI-2 in this subgroup were 18% (n = 3) and 53% (n = 9), respectively. In patients who were treated with atezolizumab/bevacizumab at ICI-2 (n = 29; 50%), ORR and DCR were 24% (n = 7) and 52% (n = 15), respectively.

### Safety

In total, 31 (53%) patients developed at least one TRAE at ICI-1 and 28 (48%) patients at ICI-2, respectively; grade 3-4 TRAEs were observed in 9 (16%) and 10 (17%) patients at ICI-1 and ICI-2, respectively. Three patients (5%) experienced grade 3-4 TRAEs at both ICI-1 and ICI-2. No treatment-related deaths were recorded. Eight (14%) and seven (12%) required systemic corticosteroid treatment at ICI-1 and ICI-2, respectively, and one patient (2%) received systemic corticosteroids at both ICI-1 and ICI-2. Four (7%) and two (3%) patients discontinued treatment due to toxicity at ICI-1 and ICI-2, respectively. TRAEs at ICI-1 and ICI-2 are summarized in Table 4.

**Table 4:** **Treatment-related adverse events at ICI-1 and ICI-2**.

	ICI-1		ICI-2	
	Any grade	Grade 3-4∗	Any grade	Grade 3∗∗
Infusion/allergic reaction	6 (10%)	1 (2%)	—	—
Arthritis/arthralgia/myalgia	5 (9%)	—	4 (7%)	—
Diarrhea/colitis	4 (7%)	1 (2%)	6 (10%)	1 (2%)
Pruritus	4 (7%)	—	2 (3%)	—
Mucositis/stomatitis	2 (3%)	1 (2%)	2 (3%)	1 (2%)
Hepatitis	2 (3%)	—	4 (7%)	3 (5%)
Dermatological	3 (5%)	—	4 (7%)	1 (2%)
Arterial hypertension	6 (10%)	2 (3%)	3 (5%)	1 (2%)
Transient ischemic attack	1 (2%)	1 (2%)	—	—
Scrotal edema	1 (2%)	1 (2%)	—	—
Amylase/lipase increase	1 (2%)	1 (2%)	—	—
Proteinuria	1 (2%)	1 (2%)	1 (2%)	1 (2%)
Nephritis	1 (2%)	—	—	—
Cough	1 (2%)	—	—	—
Sinusitis	1 (2%)	—	—	—
Thrombocytopenia	2 (3%)	—	—	—
Hypertriglyceridemia	1 (2%)	—	—	—
Palmar-plantar erythrodysesthesia	1 (2%)	—	2 (3%)	1 (2%)
Fatigue	3 (5%)	—	9 (16%)	1 (2%)
Epistaxis	1 (2%)	—	1 (2%)	—
AV block III	1 (2%)	1 (2%)	—	—
Wound healing impairment	1 (2%)	—	—	—
Hair loss	2 (3%)	—	1 (2%)	—
Nausea	—	—	1 (2%)	—
Gastritis	—	—	1 (2%)	1 (2%)
Ulcer bleeding	—	—	1 (2%)	1 (2%)
Hypothyroidism	—	—	1 (2%)	—
Hypophysitis	—	—	1 (2%)	1 (2%)
Fever	—	—	2 (3%)	—
Dysphonia	—	—	1 (2%)	—

AV, atrioventricular; ICI-1, first line of ICI treatment; ICI-2, second line of ICI treatment.

∗ No grade 5 treatment-related adverse events were recorded.

∗∗ No grade 4 or 5 treatment-related adverse events were recorded.

## Discussion

In this international, retrospective, multicenter study, an ORR of 26% and a DCR of 55% was observed in patients with HCC who received an ICI-based regimen after prior exposure to ICIs. ICI rechallenge was safe, even in patients who experienced high-grade TRAEs or required corticosteroids at ICI-1.

Several conclusions can be derived from our study. Firstly, the fact that we had to screen 994 patients in order to include 58 eligible patients (6%) suggests that ICI rechallenge is currently an uncommon practice in HCC, likely because of the lack of evidence and approval. However, there is a scientific rationale supporting the use of ICI-based therapies, particularly combinatorial regimens, in patients with primary or acquired resistance to a prior ICI regimen. For instance, upgrading from ICI monotherapy to combination treatment (*i.e*., dual ICI treatment or ICI plus tyrosine kinase inhibitors/anti-VEGF [vascular endothelial growth factor]) or using a different combination than the previous ICI regimen may restore the efficacy of immunotherapy by synergistically modulating the immunosuppressive tumor immune microenvironment through different mechanisms.[12], [13], [14] This may also be achieved by only switching the combination partner (*i.e*., TKI/anti-VEGF) while continuing with the same ICI. Indeed, in our cohort, most patients (93%) received combination therapies at ICI-2.

Secondly, patients may benefit from a second ICI regimen, even those with PD as BOR at ICI-1. In fact, ORR was similar at ICI-1 and ICI-2 in our cohort (22% *vs.* 26%), and comparable to ORRs reported for ICI-based combinations in phase III first-line trials.^4^^,^^6^ Notably, we observed responses in both patients who received ICI monotherapy and combination therapies at ICI-2, as well as in patients with primary resistance (progression as BOR) at ICI-1.

A small retrospective study reported acceptable toxicity and an ORR of 16% for dual ICI treatment (anti-CTLA-4 [cytotoxic T-lymphocyte antigen-4] plus anti-PD1 [programmed cell death 1]) in 25 patients with HCC who progressed on prior ICIs.^15^ However, in contrast to our cohort, most patients (84%) received prior PD-1 monotherapy, and only one patient was treated with atezolizumab/bevacizumab,^15^ the standard of care in systemic front-line treatment.^5^ As the most clinically relevant subgroup, 17 patients received atezolizumab/bevacizumab at ICI-1 in our cohort; in these patients, the ORR and DCR were 18% and 53%, respectively, at ICI-2. Atezolizumab/bevacizumab also led to a treatment benefit when given at ICI-2 (ORR, 24%; DCR, 52%).

Thirdly, the safety profile of the second ICI regimen was good, even in patients who experienced high-grade adverse events or required corticosteroids at ICI-1. This is in line with current recommendations suggesting that ICIs may be reinitiated – depending on the severity and site affected – once the adverse event has resolved with or without immunosuppressive treatment.^16^

Limitations of our study include the limited sample size, heterogenous population, retrospective nature, and lack of blinded response assessment at predefined intervals. Some patients received multiple lines of systemic therapy which may have led to selection of patients with less aggressive tumors and well-preserved liver function. However, the selection of a better trial population (*i.e.*, better performance status, compensated liver disease) is a *conditio sine qua non* when investigating later line treatments, and concerns not only our analysis but also large prospective studies testing second- or third-line therapies in HCC. Only patients who are alive with good performance status and well-preserved liver function are eligible for inclusion, while those with deteriorating performance status/liver function would not qualify.^17^

In conclusion, our results demonstrate that the use of ICI-based regimens after prior immunotherapy is feasible and safe, and can lead to a treatment benefit (response and stabilization) in a clinically relevant proportion of patients with HCC. These data provide a rationale for testing ICI-based therapies in patients who progressed on first-line immunotherapy in large prospective trials.

## Financial support

D.J.P. acknowledges the infrastructural support provided by Imperial Experimental Cancer Medicine Centre, Cancer Research UK Imperial Centre, the Imperial College BRC and the Imperial College Healthcare NHS Trust Tissue Bank.

## Authors’ contributions

All authors contributed either to research design (B.S. and M.P.), and/or the data acquisition (B.S., D.R., S.P., M.L., K.P., T.P., A.C., T.W.F., J.v.F., K.S., V.H., F.F., A.D., A.R.S., K.S., P.R., Bi.S., M.P.E., A.T., Ang.D., F.H., L.B., A.B.P., D.H., M.V., F.S., M.T., A.D’A., C.A.M.F., D.J.P., M.P.-R., J.-F.D., A.W., A.E.K., A.G.S., E.N.d.T., L.R., M.P.), analysis (B.S. and M.P.), or interpretation (all authors) of data. B.S. and M.P. drafted the manuscript, which was critically revised by all other authors.

## Data availability statement

The data that support the findings of this study are available from the corresponding author, MP, upon reasonable request.

## Conflicts of interest

BS received travel support from AbbVie, Ipsen and Gilead. DR has received advisory fees from Bayer and speakers fees as well as travel grants from Ipsen. He is an investigator for Bayer, BMS, Lilly, AstraZeneca and Roche. SP has nothing to disclose. ML has nothing to disclose. KP has nothing to disclose. TP received consulting fees from IQVIA and Bayer; and institutional research funding from Lilly, Roche, Bayer. AC has nothing to disclose. TWF has nothing to disclose. JVF has received advisory board fees from Roche. KS served as consultant for Ipsen and Bayer, and conducts studies for Bayer, Roche, Lilly, MSD, and BMS. VH has nothing to disclose. FF received travel support from Abbvie and Novartis, and speaker fees from Abbvie and MSD. AD has nothing to disclose. ARS has served at advisory boards and received consulting honoraria from AMGEN, AAA, Bayer, BMS, IPSEN, Lilly, Merck, MSD, Pfizer, Roche, Sanofi, and Servier. KS has nothing to disclose.

PR has nothing to disclose. BiS has nothing to disclose. MPE received consulting honoraria from BMS and MSD. AT received consulting honoraria and/or lecture fees from Bayer, IPSEN, Lilly, BMS, Eisai Novartis, Roche, Intercept, Falk, AbbVie, and Gilead. He received travel grants from IPSEN, AbbVie, and Gilead. He is an investigator for IPSEN and GILEAD. AngD received advisory board fees from Roche and BMS, and travel support from Roche and Ipsen. FH received travel support from Bayer, Abbvie, and Gilead. LB has nothing to disclose. ABP has nothing to disclose.

DH received research support from Pfizer. MV received speaker fees from Nordic Pharma, Ipsen, Merck Serono, Bayer Vital, Lilly, AstraZeneca, Merck Sharp & Dohme (MSD), Bristol-Myers Squibb (BMS), and Sirtex, advisory board fees from Roche, Ipsen, Lilly, Nordic Pharma, Bristol-Myers Squibb (BMS), Merck Sharp & Dohme (MSD), Eisai, AstraZeneca and Amgen, research grants from Sirtex. FS has nothing to disclose. MT received speaker fees from Bristol-Myers Squibb (BMS), Falk Foundation, Gilead, Intercept and Merck Sharp & Dohme (MSD); advisory board fees from Abbvie, Albireo, Boehringer Ingelheim, BiomX, Falk Pharma GmbH, GENFIT, Gilead, Intercept, Janssen, MSD, Novartis, Phenex, Regulus and Shire; travel grants from AbbVie, Falk, Gilead, and Intercept; and research grants from Albireo, CymaBay, Falk, Gilead, Intercept, MSD, and Takeda. He is also coinventor of patents on the medical use of norUDCA filed by the Medical University of Graz. ADA received travel support and consultancy fees from Roche. CAMF has nothing to disclose. DJP received lecture fees from ViiV Healthcare, Bayer Healthcare, BMS, Roche, Eisai, Falk Foundation, travel expenses from BMS and Bayer Healthcare; consulting fees for Mina Therapeutics, EISAI, Roche, DaVolterra, Mursla, Exact Sciences and Astra Zeneca; research funding (to institution) from MSD and BMS. MPR is advisor/consultant for Astra Zeneca, Bayer, BMS, Eisai, Ipsen, Lilly, MSD, and Roche; he served as a speaker for Bayer, Eisai, Ipsen, Lilly, and Roche; he is an investigator for Bayer, BMS, Eisai, Exelixis, Lilly, and Roche. JFD received compensations as a member of scientific advisory boards of Abbvie, Bayer, Bristol–Myers Squibb, Falk, Galapagos, Genfit, Genkyotex, Gilead Sciences, HepaRegenix, Intercept, Lilly, Merck, and Novartis. AW received compensations as a member of scientific advisory boards for BMS, Wako and Sanofi. He served as a speaker for Leo Pharma, Eisai, Ipsen and Roche and received travel support from Merck and Servier. AEK has received consulting fees from Abbvie, AstraZeneca, Bayer, CymaBay, Escient, FMC, Gilead, GSK, Guidepoint, Intercept, Mirum, Medscape, MSD, Myr, Viofor; lecture fees from Abbvie, AOP Orphan, Bayer, BMS, CMS, CymaBay, Eisai, Falk, Gilead, GSK, Intercept, Janssen, Newbridge, Novartis, Lilly, MSD, Zambon; and institutional research funding from Intercept. AGS served on advisory boards and as a consultant for Genentech, AstraZeneca, Eisai, Bayer, Exelixis, TARGET RWE, FujiFilm Medical Sciences, Glycotest, Exact Sciences, GRAIL, and Freenome. ENDT has served as a paid consultant for AstraZeneca, Bayer, BMS, EISAI, Eli Lilly & Co, MSD, Mallinckrodt, Omega, Pfizer, IPSEN, Terumo and Roche. He has received reimbursement of meeting attendance fees and travel expenses from Arqule, Astrazeneca, BMS, Bayer, Celsion and Roche, and lecture honoraria from BMS and Falk. He has received third-party funding for scientific research from Arqule, AstraZeneca, BMS, Bayer, Eli Lilly, and IPSEN and Roche. LR has received consulting fees from Amgen, ArQule, AstraZeneca, Basilea, Bayer, BMS, Celgene, Eisai, Exelixis, Genenta, Hengrui, Incyte, Ipsen, IQVIA, Lilly, MSD, Nerviano Medical Sciences, Roche, Sanofi, Servier, Taiho Oncology, Zymeworks; lectures fees from AbbVie, Amgen, Bayer, Eisai, Gilead, Incyte, Ipsen, Lilly, Merck Serono, Roche, Sanofi, Servier; travel expenses from AstraZeneca; and institutional research funding from Agios, ARMO BioSciences, AstraZeneca, BeiGene, Eisai, Exelixis, Fibrogen, Incyte, Ipsen, Lilly, MSD, Nerviano Medical Sciences, Roche, Zymeworks.

MP is an investigator for Bayer, BMS, Eisai, Ipsen, Lilly, and Roche; he received speaker honoraria from Bayer, BMS, Eisai, Lilly, MSD, and Roche; he is a consultant for Bayer, BMS, Eisai, Ipsen, Lilly, MSD, and Roche; he received travel support from Bayer, BMS, and Roche.

Please refer to the accompanying ICMJE disclosure forms for further details.
