# Supplementary material for: Efficacy and safety of immune checkpoint inhibitor rechallenge in individuals with hepatocellular carcinoma
Source: JHEP Rep. 2022 Oct 27;5(1):100620. doi: 10.1016/j.jhepr.2022.100620 (PMC9791167; doi:10.1016/j.jhepr.2022.100620)
Supplement: Multimedia component 1 [file mmc1.pdf]

Efficacy and safety of immune checkpoint inhibitor rechallenge in individuals with hepatocellular carcinoma

Bernhard Scheiner, Daniel Roessler, Samuel Phen, Mir Lim, Katharina Pomej, Tiziana Pressiani, Antonella Cammarota, Thorben W. Fründt, Johann von Felden, Kornelius Schulze, Vera Himmelsbach, Fabian Finkelmeier, Ansgar Deibel, Alexander R. Siebenhüner, Kateryna Shmanko, Pompilia Radu, Birgit Schwacha, Matthias P. Ebert, Andreas Teufel, Angela Djanani, Florian Hucke, Lorenz Balcar, Alexander B. Philipp, David Hsiehchen, Marino Venerito, Friedrich Sinner, Michael Trauner, Antonio D'Alessio, Claudia A.M. Fulgenzi, David J. Pinato, Markus Peck-Radosavljevic, Jean-François Dufour, Arndt Weinmann, Andreas E. Kremer, Amit G. Singal, Enrico N De Toni, Lorenza Rimassa, Matthias Pinter

Table of contents

Table S1..2

Table S2..3

**Table S1.** Agents used at ICI-1 and ICI-2		
**Agents**	**ICI-1**	**ICI-2**
Atezolizumab/bevacizumab	17 (29%)	29 (50%)
Nivolumab	15 (26%)	–
Pembrolizumab	9 (16%)	3 (5%)
Pembrolizumab/bavituximab	7 (12%	–
Nivolumab/lenvatinib	3 (5%)	–
Pembrolizumab/lenvatinib	2 (3%)	2 (3%)
INCB086550	2 (3%)	–
Nivolumab/ipilimumab	1 (2%)	12 (21%)
Atezolizumab/cabozantinib	1 (2%)	3 (5%)
Pembrolizumab/cabozantinib	1 (2%)	–
Pembrolizumab/regorafenib	–	3 (5%)
Cemiplimab/SAR439459	–	3 (5%)
Atezolizumab/lenvatinib	–	1 (2%)
Ipilimumab	–	1 (2%)
Pembrolizumab/bevacizumab	–	1 (2%)
Abbreviations: ICI-1, first line of ICI treatment; ICI-2, second line of ICI treatment		

Table S2. Agents used at ICI-2 and best overall response at ICI-2 in the subgroup of patients who received atezolizumab plus bevacizumab at ICI-1 (n=17)

	**Best overall response, n**	
**Agents at ICI-2 (n=17, 100%)**	**CR or PR**	**SD**
Nivolumab/ipilimumab (n=8, 47%)	2	1
Atezolizumab/cabozantinib (n=3, 18%)	-	3
Pembrolizumab/lenvatinib (n=1, 6%)	-	-
Atezolizumab/lenvatinib (n=1, 6%)	-	1
Cemiplimab/SAR439459 (n=1, 6%)	-	-
Pembrolizumab/regorafenib (n=1, 6%)	-	1
Pembrolizumab (n=1, 6%)	1	-
Ipilimumab (n=1, 6%)	-	-

Abbreviations: CR, complete response; ICI-1, first line of ICI treatment; ICI-2, second line of ICI treatment; PR, partial response; SD, stable disease
